# Changes in Brain Oscillatory Dynamics in Elderly Adults as a Consequence of Natural Aging

**DOI:** 10.1002/alz70856_104444

**Published:** 2025-12-26

**Authors:** Lucila Capurro, Michael Radloff, Luis Ignacio Brusco, Rodrigo Ramele, Cecilia Forcato

**Affiliations:** ^1^ Technological Institute of Buenos Aires (ITBA), Buenos Aires, Buenos Aires, Argentina; ^2^ Department of Health Psychology, Institute for Psychology, University of Klagenfurt, Klagenfurt, Kärnten, Austria; ^3^ University of Buenos Aires, Buenos Aires, Argentina; ^4^ ALZAR ‐ Argentine Alzheimer's Association, Buenos Aires, CABA, Argentina; ^5^ National Scientific and Technical Research Council (CONICET), Buenos Aires, Argentina; ^6^ Universidad de Buenos Aires, Buenos Aires, Argentina

## Abstract

**Background:**

The oscillatory nature of slow waves during non‐rapid eye movement (NREM) sleep has recently been proposed as crucial for the glymphatic system, facilitating the clearance of metabolic waste from the brain. While aging‐related reductions in slow wave quantity and amplitude are well‐documented and linked to this cleansing function, we propose that the rhythmic dynamics in which slow waves occur may also play a critical role.

**Method:**

Thus, we introduce a novel classification of slow waves based on their temporal dynamics, categorizing them into isolated waves and oscillation trains. Using overnight EEG recordings from young and elderly adults, we compared the proportions of these wave types. Additionally, we analyzed train composition, including the proportion of slow waves that initiate a train (lead waves) and the lengths of the oscillation trains (number of consecutive slow waves initiated by one lead wave).

**Result:**

Our results revealed that elderly adults exhibited a higher prevalence of isolated waves and a lower proportion of oscillation trains. Moreover, while elderly adults showed a higher proportion of lead waves, their oscillation trains were significantly shorter compared to those of young adults.

**Conclusion:**

We propose that natural aging may result in a less oscillatory brain state, characterized by a diminished ability to produce sustained, periodic oscillations. This diminished rhythmicity could impair cerebrospinal fluid pulsation, potentially reducing the brain's ability to efficiently clear pathogenic substances during sleep. Given the established link between impaired glymphatic clearance and neurodegenerative diseases such as Alzheimer's, this diminished capacity to sustain slow wave trains may contribute to age‐related decline in neurological functioning.

